# Anti-Heartburn Effects of Sugar Cane Flour: A Double-Blind, Randomized, Placebo-Controlled Study

**DOI:** 10.3390/nu12061813

**Published:** 2020-06-18

**Authors:** Jeffrey M. Beckett, Neeraj K. Singh, Jehan Phillips, Krishnakumar Kalpurath, Kent Taylor, Roger A. Stanley, Rajaraman D. Eri

**Affiliations:** 1 School of Health Sciences, University of Tasmania, Launceston, TAS 7250, Australia; Jeffrey.Beckett@utas.edu.au (J.M.B.); Neeraj.Singh@utas.edu.au (N.K.S.); 2 First Point Healthcare, Launceston, TAS 7250, Australia; jehanp@westnet.com.au; 3 Mersey Community Hospital, Latrobe, TAS 7307, Australia; krishna.kalpurath@ths.tas.gov.au; 4 KFSU Pty Ltd., Ayr, QLD 4807, Australia; ktaylor@kfsu.com.au; 5 Centre for Food Innovation, Tasmanian Institute of Agriculture, University of Tasmania, Launceston 7250, Australia; roger.stanley@utas.edu.au

**Keywords:** gastroesophageal reflux disease, heartburn, regurgitation, sugarcane flour, dietary fiber

## Abstract

Gastroesophageal reflux disease (GERD) affects approximately 20% of Australians. Patients suffer a burning sensation known as heartburn due to the movement of acidic stomach content into the esophagus. There is anecdotal evidence of the effectiveness of prebiotic sugarcane flour in controlling symptoms of GERD. This pilot study aimed to investigate the effectiveness of a prebiotic sugarcane flour in alleviating symptoms in medically-diagnosed GERD patients. This pilot study was a single center, double-blinded, placebo-controlled randomized trial conducted on 43 eligible participants. The intervention group (*n* = 22) were randomized to receive 3 g of sugarcane flour per day, and the control group (*n* = 21) received 3 g of cellulose placebo per day. Symptoms of gastroesophageal reflux disease were assessed before and after three weeks treatment using the validated Gastroesophageal Reflux Disease-Health Related Quality of Life questionnaire (GERD-HRQL). After three weeks there were significant differences in symptoms for heartburn, regurgitation, and total symptoms scores (*p* < 0.05) between the sugarcane flour and placebo. Mean GERD-HRQL scores increased in the placebo group for regurgitation (mean increase 1.7; 95% CI 0.23 to 3.2; *p* = 0.015) and total symptom scores (2.9; 95% CI 0.26 to 5.7; *p* = 0.033). In contrast, there were significant reductions in heartburn (mean decrease −2.2; 95% CI −4.2 to −0.14; *p* = 0.037) and total symptom scores (−3.7; 95% CI −7.2 to −0.11; *p* = 0.044) in the intervention group. This pilot study has shown significant positive effects of sugarcane flour in the reduction of GERD symptoms, and a larger randomized controlled trial is warranted.

## 1. Introduction

Gastroesophageal reflux disease (GERD) is symptomatically referred to as heartburn or reflux disease, where the stomach content can travel backwards towards the esophagus leading to gastric content attack on the tissue lining. The primary symptoms of GERD are heartburn and regurgitation, but individuals may also suffer chest pain, nausea, bloating, belching, and water brash [1]. Major contributing factors in the pathogenesis of GERD are reported as reduced esophageal motility, low esophageal clearance, and acidification of the esophagus [2,3]. There is a large body of evidence that significant morbidity is associated with GERD, which is suggested to affect around 20% of Australians [4]. GERD can cause drastic reductions in quality of life including lack of quality sleep, dysphagia, and in severe cases, esophageal cancer [5,6]. Current GERD treatments mainly consist of lifestyle changes, use of acid antisecretory drugs and surgery. Proton pump inhibitors (PPI) are generally used for long term maintenance and treatment of reflux esophagitis [1,7,8]. While PPIs work well in over half of the GERD patients, there is a refractory group that needs more effective alternative therapies [9]. Additionally, recent studies have begun to raise concerns regarding the long-term effects correlated to the use of PPI’s and the need for greater consideration and study of their risk profile [10].

Low fiber intake has been associated with decreased stomach and gut motility [11], and subsequently an increase in gastric emptying time and gastric overdistension, which are known risk factors of gastroesophageal reflux [12]. Additionally, development of hiatal hernia is an anatomical abnormality. When associated with low fiber consumption hiatal hernia may adversely affect the anti-reflux barrier [2,13,14]. Lifestyle changes, such as an increase in the consumption of dietary fiber, have been shown to be effective in decreasing esophageal reflux episodes and their severity by enhancing the rate of gastric emptying and improving gastric acid reduction [2,15]. There is also a hypothesized mechanism that dietary fiber can bind to the nitric oxide content of the food and result in a reduction of the gas pressures experienced by the lower esophageal sphincter [2].

Prebiotic whole plant sugarcane flour (PSCF) is a propriety product made by KFSU Ltd. (K-fibre™; Ayr, QLD, Australia) [16] from freshly harvested sugar cane. While most of the sucrose is removed by a water diffusion process, PSCF retains much of its cellular structures and biochemical complexity. These include soluble and insoluble fiber fractions comprised of cell wall attached glycan chains that can be fermented by intestinal flora. These complex fibrous structures are reported to be more uniformly fermented throughout the entire digestive tract including the hindgut, in contrast to isolated soluble fiber supplements which are generally rapidly fermented [17].

Anecdotal evidence from user feedback suggested that PSCF was effective in controlling the symptoms of GERD. There are no similar reports of whole plant fiber being effective for relief of heartburn symptoms. PSCF has however, recently been shown, in conjunction with probiotic supplementation, to have beneficial effects in an inflammatory bowel disease mouse model [18]. Therefore, the purpose of this study was to carry out a pilot trial to test the efficacy of this sugarcane flour product in controlling of symptoms in medically diagnosed GERD patients.

## 2. Materials and Methods

This pilot study was conducted between June and October 2019. The study was approved by the Human Research Ethics Committee (Tasmania) Network, Australia (Reference H0017109) and all participants provided written informed consent. The study was registered with the Australian Clinical Trials Registry (ACTRN12619001204134) [19].

Men and women were enrolled as patients of a medical clinic in Launceston, Tasmania, Australia. Eligible participants were people aged 18 to 75 years, with heartburn and/or regurgitation at least three times a week during the seven-day run-in period prior to randomization; able to read, understand, and complete the study questionnaire and records; able to understand the study procedures and sign informed consent; and able to comply with all study requirements. Exclusion criteria were: Diagnosis of Barrett’s esophagus, non-erosive reflux disease, erosive esophagitis grades A–D, or peptic stricture on endoscopy, previous upper gastrointestinal surgery, clinically significant underlying comorbidity, *Helicobacter pylori* positive, clinically significant GI bleeding within the last three months, esophagitis not related to acid reflux, bleeding disorder, Zollinger–Ellison Syndrome, achalasia, esophageal varices, duodenal/gastric ulcer, and upper gastrointestinal malignancy.

This was a single center double blinded placebo-controlled study to test the effect of PSCF consumption on GERD symptoms. No similar studies on the effect of PSCF on GERD symptoms were found in a literature review, so a sample size calculation was not possible. This study was therefore conducted as a pilot study with a target sample size of 40. Participants were randomly assigned into two treatment groups using a randomization table produced by the random number generation function in Microsoft Excel by a person not involved in data collection. Participants and researchers assessing participants were blinded to the intervention. The intervention materials were very similar in appearance and taste and provided in numbered containers. Group 1 participants received PSCF intervention, while Group 2 participants received a cellulose placebo. All participants were given a 150 g container of the PSCF intervention (K-fibre™; Ayr, QLD, Australia) or the cellulose placebo (Regenerated cellulose fiber; PF100, Interfiber, Poland) with instructions on use. Participants were instructed to stir one heaped measuring spoon of PSCF or placebo into a glass of water and consume after food each morning and night), ensuring it was consumed immediately in suspended form. Due to density differences, a 5 mL dosing spoon for PSCF and 7.5 mL dosing spoon for cellulose placebo was used to deliver 1.5 g, and therefore a total of 3 g per day. After three weeks of treatment participants returned to the clinic for assessment and returned the unused portion of the fiber to validate consumption.

Symptoms of GERD were assessed before and after treatment using the validated Gastroesophageal Reflux Disease-Health Related Quality of Life (GERD-HRQL) questionnaire [20]. This questionnaire provides a quantitative method for measuring symptom severity in GERD. It assesses both individual heartburn (heartburn score, maximum of 30) and regurgitation symptoms (regurgitation score, maximum of 30), and provides a composite score (maximum of 75). The questionnaire is comprised of nine questions with responses ranging from 0 to 5, where 0 = no symptom and 5 = symptoms are incapacitating to daily activities. The time required to administer the questionnaire was approximately 2 min. The questionnaire was administered by the participating clinician at enrolment and again after the completion of three weeks of intervention. 

Adverse events were monitored throughout the study by the participating clinician. Participants were not allowed to consume any other fiber products or medications for the treatment of GERD symptoms such as PPI and antacids.

### Statistical Analysis

Continuous variables were reported as mean and standard deviations. Normality of data was determined using D’Agostino-Pearson normality test. Baseline comparisons were performed using unpaired t-test and Mann–Whitney test. Changes within the two groups were assessed using paired t-test and Wilcoxon matched-pairs test. Categorical variables were analyzed using Fisher’s exact test. Statistical analysis was performed using GraphPad Prism 7.0 (GraphPad Software, La Jolla, CA, USA). A *p* value < 0.05 was considered statistically significant.

## 3. Results

### 3.1. Demographics and Baseline

**Figure 1:**
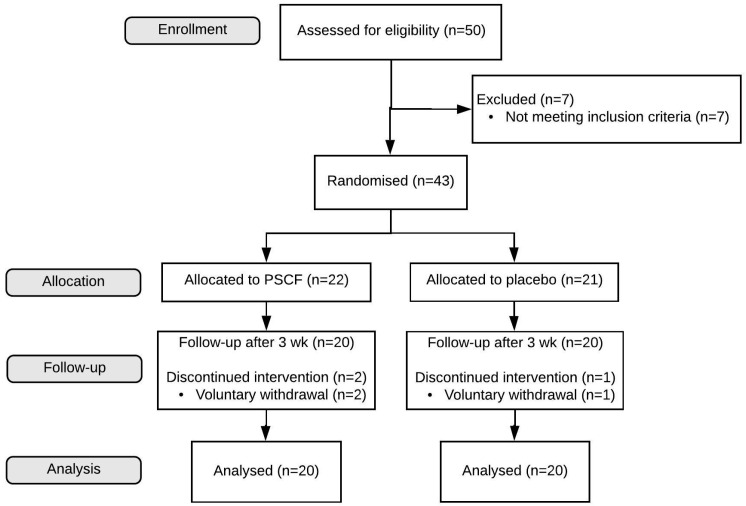
Flow chart representing the pilot study.

Fifty people consented to participate in the study; seven were excluded due to not meeting the inclusion criteria, leaving 43 participants to be randomized (Figure 1). During follow-up, three participants (one from placebo group; two from the PSCF intervention group) voluntarily withdrew from the study. The mean (±SD) age of participants (26 females, 14 males) that completed the study was 46.0 ± 12.6 years (range: 21–74 years) and BMI was 32.6 ± 8.7 kg/m^2^. Fifteen participants were current smokers (seven in placebo group, eight in PSCF intervention group) (Table 1). Of the participant demographics, only age was significantly different (*p* = 0.009) between the placebo and PSCF group. GERD-HRQL questionnaire scores between placebo and PSCF intervention groups at baseline (Table 1) were significantly higher in the PSCF group (*p* = 0.039) for the regurgitation score. The intervention formulations were well tolerated, with no reports of adverse effects among study participants after three weeks.

### 3.2. Changes after Three Weeks

**Figure 2:**
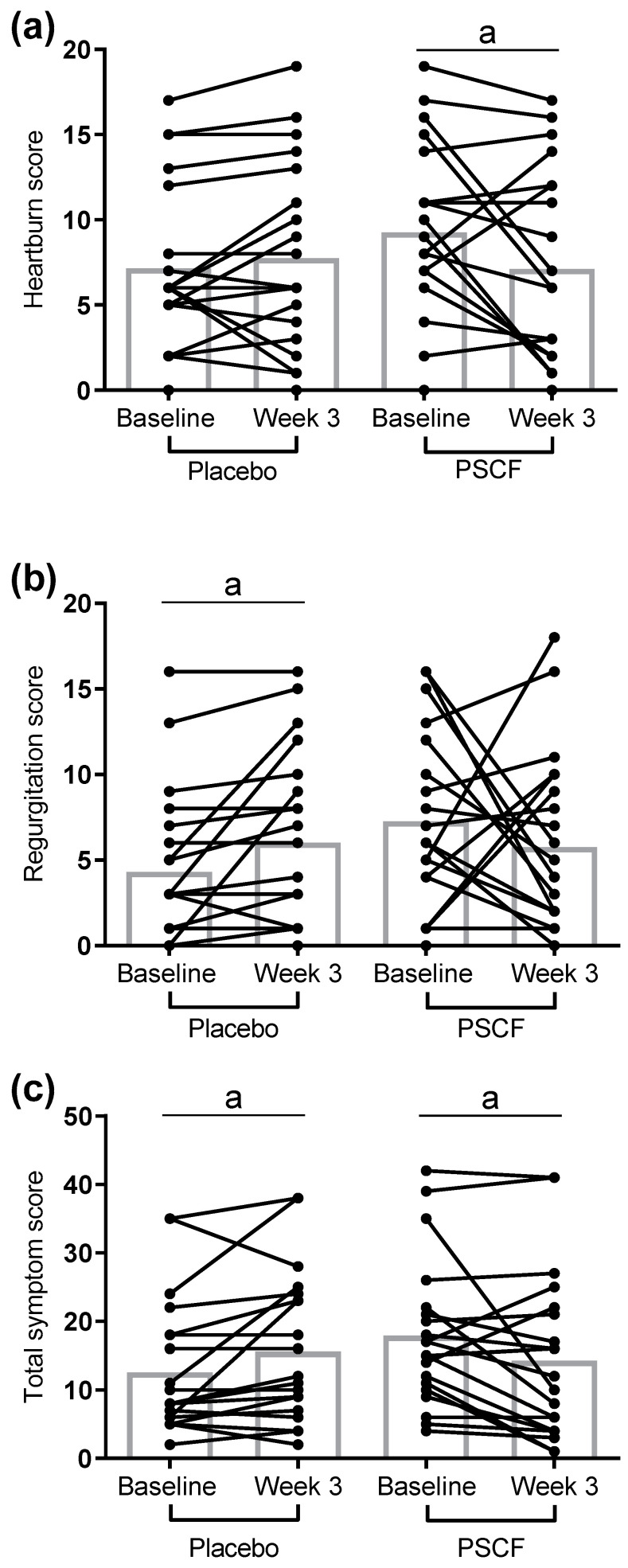
Individual participant changes in self-reported GERD-HRQL symptom scores after 3 weeks of intervention. Heartburn symptom score (**a**), regurgitation symptom score (**b**), and total symptom score (**c**) changes after 3 weeks of intervention. Bar charts show mean values. ^a^ *p* < 0.05. GERD-HRQL: Gastroesophageal Reflux Disease-Health Related Quality of Life; PSCF: prebiotic whole plant sugar cane flour.

After three weeks of intervention, there were significant differences (*p* < 0.05) between placebo and PSCF in the classification of symptoms (improved, unchanged or worsened) for individual heartburn and regurgitation symptom scores and total symptom score (Table 2). Mean GERD-HRQL questionnaire scores in the placebo group increased (Figure 2), with regurgitation symptom score (1.7, 95% CI 0.23 to 3.2; *p* = 0.015) and the total symptom score (2.9; 95% CI 0.26 to 5.7; *p* = 0.033) reaching statistical significance. In contrast, in the PSCF intervention group, mean GERD-HRQL questionnaire scores decreased after 3 weeks. In this case, changes in both heartburn symptom score (−2.2; 95% CI −4.2 to −0.14; *p* = 0.037) and total symptom score (−3.7; 95% CI −7.2 to −0.11; *p* = 0.044) were statistically significant.

## 4. Discussion

Our investigation into the effects of sugar cane flour through a pilot randomized, placebo- controlled trial showed a significant average reduction in self-reported GERD symptoms after 3 weeks of consumption. PSCF consumption in this trial showed an overall reduction in both heartburn and regurgitation scores, indicating acid reflux symptom relief aligned with prior informal reports by product consumers. However, while improvements to symptoms were shown by most subjects, heartburn, regurgitation, and total score still worsened with PSCF in 25, 35, and 30% respectively noting that the equivalent figures for the placebo were 55, 50, and 65%. As the mechanism for alleviation of GERD by fiber is not yet understood, speculation as to why subjects reacted differently can include other dietary factors that may be antagonistic to fiber action, inherent problems associated with neuro-hormonal control mechanism that regulates satiety and gastric emptying and the diversity of gut microbiome.

Regardless of why some subjects improved, and other did not, a number of epidemiological studies have clearly pointed to a negative correlation between dietary fiber intake and frequency of reflux [21,22]. However, studies directly involving dietary interventions for the treatment or symptom alleviation in GERD is a recent concept. Countering GERD pathogenesis has conventionally been limited to treatments targeting the gastroesophageal junction (lower esophageal sphincter) and stomach acid reduction. The major therapies, such as antacids and PPI drugs, are aimed at reducing acid production in the stomach or reducing gastric pressure and increased gastric emptying to prevent flow of acid to esophagus [23,24,25]. The mode by which the PSCF affected the perceptions of GERD symptoms cannot be deduced from this trial. While acute rapid relief response to PSCF use has been informally reported, there may be a cumulative effect that could be determined by multiple sequential assessments. The observed reduction in GERD activity may also be related to the amount and type of fiber intake. There have been two previous studies directly involving fiber products. Firstly, DiSilvestro et al., in 2011 [26] showed that two weeks intervention with 4 g of fenugreek fiber product daily reduced GERD symptoms, and recently Morozov et al., [2] demonstrated that a fiber enriched diet using 5 g psyllium (Plantago ovata husk fiber) daily reduced reflux symptoms and esophageal motility. In these studies, the fibers used highly absorb water to form viscous soluble products with the implication that viscosity and surface coating could be mechanistic in activity. Viscosity and gut mucosal adherence may also influence barrier functions improving barrier properties to enzymatic and chemical influences that can cause inflammation and ulceration. For example, psyllium and related viscous products can decrease the rate of glucose adsorption. For GERD relief, prior studies have considered fibers with viscous properties that could be inferred to have barrier enhancing effects in the stomach and possibly the esophagus [2]. In this study the PSCF has a water adsorptive capacity of around 6:1 w/w and viscosity is relatively low, making a barrier function mechanism unlikely.

At the current stage, the further definition of a mechanism for how PSCF might be imparting anti-reflux activity observed in our study can only be based on indirect evidence. Gut motility and gastric emptying may be influenced by the presence of the particles [27]. Particle size is known to effect gastric emptying; emptying being accelerated by small particle sizes [28]. Large bran particles cause increased heartburn limiting its uses against constipation. The large particles cause significant delay in gastric emptying compared to small bran particles [29]. In this study PSCF is an insoluble material made up a small fragments of ground sugar cane stems with a mean size of 90.6 µm, similar to the placebo control with a mean of 100 µm. PSCF, however, has microscopically visible sharp shape features due to the presence of linear fragments associated with xylem and phloem tubes in vascular bundles of the cane stem that conduct water and sugars to and from the leaves. These needle-like features are assumed to be the cause of the throat catch issues which can give an irritation through the mucus membranes in a manner related to sharp oxalate needles in kiwifruit pulp [30]. In contrast the placebo used in this study was regenerated cellulose, of the type used for excipient applications in the formation of medicinal tablets to swallow. It has a more rounded particle without the sharp features of the PSCF but is of a similar size, appearance, and taste. There is therefore a possibility that the shape and physical interaction of the PSCF with mucosal surfaces is improving either stomach motility, stomach emptying, or tightening the upper esophageal sphincter muscles.

Fiber intake has the ability to reduce the overall food consumption of an individual by reducing the GI of the meal, in turn reducing the quantity of meal consumed by a person and by acting at the satiety center to prolong time between meals. This can occur through modulating the bacterial products to act on the gut–brain axis for satiety and gastric emptying control. Both these aspects are likely to assist in reducing gastric pressure. Additionally, whole plant fibers are known to increase gastric emptying [31], hence, increased gastric emptying may reduce the gastric pressure and the retrograde flow of stomach contents to esophagus. However, fiber intake recommendations, as given in the Nutrient Reference Values for fiber intake in Australia are 25–30 g/day for adults where AI is set at the median for dietary fiber intake in Australia (https://www.nrv.gov.au/nutrients/dietary-fibre). In this study the amount of extra fiber consumed was much less, at around 3 g/day, and would not have been a major increase in fiber intake. Alternative mechanisms that may be applicable at the relatively low doses of PSCF include binding of nitric oxide. A number of studies have pointed to nitric oxide as a major player coordinating esophageal peristalsis [32]. Reducing gastroesophageal reflux activity through the action of binding nitric oxide to the lignin or silica components of the soluble fiber can also improve esophageal peristalsis. Further studies on interaction between fiber and nitric oxide at the level of lower esophageal sphincter (LES) could provide a better explanation.

Our study has a number of limitations that are attributable to certain difficulties in conducting clinical trials of this nature. Major limitations include the dependence of self-reported GERD questionnaire, without clinical measurements for GERD symptoms; lack of data on diet in study subjects and the small number of subjects in each group. These limitations, however, are inherent to the pilot study nature of this research.

In summary, our study has shown significant benefit of fiber supplementation for reducing GERD symptoms. Sugar cane whole plant fiber used in the study was well tolerated and showed GERD symptom alleviation within 3 weeks. While the exact mechanism is not fully understood, the clinical benefit indicatively demonstrated warrants a larger multi-center randomized controlled trial with dose escalation and longer duration intervention.

## Figures and Tables

**Table 1:** Participant characteristics at baseline of pilot study.

Variable	Placebo (*n* = 20)	PSCF (*n* = 20)	*p* Value
Gender, *n* (%)			
Male	6 (30)	8 (40)	
Female	14 (70)	12 (60)	
Age, years	41.0 ± 12.9	51.1 ± 10.4	0.009
Height, cm	167 ± 7	169 ± 7	0.425
Weight, kg	91 ± 23	90 ± 24	0.880
Body mass index, kg/m^2^	32 ± 9	33 ± 9	0.878
Smoking, yes %	35	40	1.000
GERD-HRQL scores			
Heartburn	7.2 ± 4.8	9.3 ± 5.1	0.187
Regurgitation	4.3 ± 4.5	7.3 ± 5.1	0.039
Total	12.6 ± 10.0	17.9 ± 10.7	0.109

GERD-HRQL: Gastroesophageal Reflux Disease-Health Related Quality of Life; PSCF: prebiotic whole plant sugar cane flour. Data presented as mean ± SD.

**Table 2:** Classification of self-reported symptoms after three weeks of intervention.

Symptom	Placebo (*n* = 20)	PSCF (*n* = 20)	*p* Value
Heartburn, *n* (%)			
Improved	5 (25)	13 (65)	0.039
Unchanged	4 (20)	2 (10)	
Worsened	11 (55)	5 (25)	
Regurgitation, *n* (%)			
Improved	1 (5)	11 (55)	0.001
Unchanged	9 (45)	2 (10)	
Worsened	10 (50)	7 (35)	
Total score, *n* (%)			
Improved	4 (20)	13 (65)	0.015
Unchanged	3 (15)	1 (5)	
Worsened	13 (65)	6 (30)	

PSCF: Prebiotic whole plant sugar cane flour.
